# Chlorpromazine Induces Basolateral Aquaporin-2 Accumulation via F-Actin Depolymerization and Blockade of Endocytosis in Renal Epithelial Cells

**DOI:** 10.3390/cells9041057

**Published:** 2020-04-23

**Authors:** Richard Bouley, Naofumi Yui, Abby Terlouw, Pui W. Cheung, Dennis Brown

**Affiliations:** 1 Program in Membrane Biology and Nephrology Division, Massachusetts General Hospital and Harvard Medical School, Boston, MA 02114 USA; bouley.richard@mgh.harvard.edu (R.B.); nyuikid@tmd.ac.jp (N.Y.); abbyterlouw@gmail.com (A.T.); Pui.cheung@mgh.harvard.edu (P.W.C.); 2 Department of Nephrology, Tokyo Medical and Dental University, Tokyo 113-8519, Japan

**Keywords:** water-channel, aquaporin, LLC-PK1 kidney cells, forskolin, vasopressin

## Abstract

We previously showed that in polarized Madin–Darby canine kidney (MDCK) cells, aquaporin-2 (AQP2) is continuously targeted to the basolateral plasma membrane from which it is rapidly retrieved by clathrin-mediated endocytosis. It then undertakes microtubule-dependent transcytosis toward the apical plasma membrane. In this study, we found that treatment with chlorpromazine (CPZ, an inhibitor of clathrin-mediated endocytosis) results in AQP2 accumulation in the basolateral, but not the apical plasma membrane of epithelial cells. In MDCK cells, both AQP2 and clathrin were concentrated in the basolateral plasma membrane after CPZ treatment (100 µM for 15 min), and endocytosis was reduced. Then, using rhodamine phalloidin staining, we found that basolateral, but not apical, F-actin was selectively reduced by CPZ treatment. After incubation of rat kidney slices in situ with CPZ (200 µM for 15 min), basolateral AQP2 and clathrin were increased in principal cells, which simultaneously showed a significant decrease of basolateral compared to apical F-actin staining. These results indicate that clathrin-dependent transcytosis of AQP2 is an essential part of its trafficking pathway in renal epithelial cells and that this process can be inhibited by selectively depolymerizing the basolateral actin pool using CPZ.

## 1. Introduction

Vasopressin (VP)-mediated apical accumulation of aquaporin 2 (AQP2) is crucial for water reabsorption and urine concentration in the kidney collecting duct [1,2,3,4]. To initiate this process, VP induces changes in the phosphorylation state of AQP2 at several serine residues [5,6]. Phosphorylation of Ser-256, -264, and -269 is greatly increased, [7,8], whereas Ser-261 phosphorylation is decreased by VP stimulation [9]. In nonstimulated, baseline conditions, AQP2 is continuously targeted to the plasma membrane from which it is rapidly internalized by constitutive clathrin-mediated endocytosis, maintaining an intrinsic balance that favors the cytoplasmic, vesicular distribution of AQP2 independent of its phosphorylation state [10,11,12]. VP offsets the balance toward AQP2 membrane accumulation by increasing AQP2 exocytosis and decreasing AQP2 endocytosis [13,14,15]. Modulation of the endocytic machinery plays a major role in VP-regulated AQP2 apical accumulation, and the phosphorylation state of AQP2 is essential for the interaction between AQP2 with endocytosis-regulating molecules [16,17,18,19]. In addition, AQP2 selectively and catalytically depolymerizes apical F-actin through interaction with actin-related molecules, e.g., tropomyosin 5b [20,21]. While the molecular mechanisms of water channel accumulation in the apical plasma membrane of collecting duct cells have been of particular interest, the regulation of basolateral AQP2 has also been investigated in several studies [22,23,24,25,26]. In the cortical connecting segment, basolateral AQP2 is usually readily detectable [23], and AQP2 has varying levels of basolateral expression in collecting duct principal cells from kidney cortex to medulla, with the most obvious basolateral expression in the inner medulla. In MDCK cells, derived from canine kidney, transfected AQP2 usually accumulates in the apical membrane upon intracellular cAMP elevation. However, AQP2 instead concentrates on the basolateral membrane after forskolin (FK) treatment under hypertonic conditions [26]. Importantly, Sec6, Sec8, and RalA, all components of the exocyst, which targets proteins to the basolateral plasma membrane [27], were associated with AQP2-containing vesicles upon mass spectrometry analysis [28]. Furthermore, we showed that AQP2 is necessary for cell migration and tubule morphogenesis through its RGD-regulated interaction with basolateral integrin-β1 [29]. These previous findings all pointed to the existence of an AQP2 basolateral targeting pathway, and indeed we demonstrated the existence of a transcytotic pathway for AQP2 in MDCK cells by blocking basolateral AQP2 during its transit through this membrane domain using low temperature [30]. After initial basolateral targeting, AQP2 is retrieved into Rab5-positive vesicles, then undertakes microtubule-dependent transcytosis to apical recycling vesicles [30]. This novel trafficking concept is supported by an apical plasma membrane proteomics study using mpkCCD cells, in which Mal2, a molecule important for transcytosis, was shown to be greatly increased by vasopressin [31]. At present, while the concept of AQP2 transcytosis is gaining traction [32], evidence that this process also occurs in animal kidneys, independently of cold shock, is now required.

In this study, we used chlorpromazine (CPZ), a drug that blocks endocytosis of the Na-K-2Cl cotransporter (NKCC) in MDCK cells [33], to examine its effect on AQP2 subcellular distribution in both MDCK cells and rat kidney slices. Our results validate the existence of AQP2 transcytosis and reveal the importance of basolateral F-actin and clathrin-mediated endocytosis in this process. 

## 2. Materials and Methods

Forskolin (FK), chlorpromazine hydrochloride (CPZ), latrunculin B (LtB), methyl-β-cyclodextrin (MBCD), rhodamine phalloidin (phalloidin tetramethylrhodamine B isothiocyanate), rhodamine-tagged transferrin (Rho-Tf), and mouse anti-β-tubulin antibody (T8328) were purchased from Sigma Aldrich (St. Louis, MO, USA). Alexa Fluor 647-tagged wheat germ agglutinin was purchased from Invitrogen (Carlsbad, CA, USA). Goat anti-AQP2 antibody (sc9882, C-17) was purchased from Santa Cruz (Santa Cruz, CA, USA). Mouse anti-clathrin antibody (610499) was purchased from BD Transduction Labs (San Jose, CA, USA). Rabbit polyclonal antiphospho-AQP2 antibodies were kindly provided by Dr. Mark Knepper (NIH, Bethesda, MD, USA). 

### 2.1. Cell Culture

Madin–Darby canine kidney (MDCK) cells stably expressing rat AQP2 (AQP2-MDCK cells) were generated as described previously [34], grown at 37 °C (5% CO_2_) in 10% FBS DMEM containing penicillin/streptomycin (PS), and passaged using TrypLE (GIBCO-BRL, Carlsbad, CA, USA). 

### 2.2. Western Blotting

MDCK cells were grown on six well plates for 3 days, and incubated overnight with 50 µM indomethacin to reduce endogenous cAMP levels as previously described [35], and then were subjected to drug treatment. Cells were lysed in lysis buffer (150 mM NaCl, 20 mM Tris-HCl, 5 mM EDTA, and 1% Triton X-100) supplemented with Complete Mini (CM) protease inhibitor (1 tablet/40 mL), containing 10 mM sodium fluoride (NaF). The lysates were rotated for 30 min at 4 °C and then centrifuged for 10 min at 6000 g. The protein concentration was determined using a BCA kit (Pierce, Rockford, IL, USA). Samples were diluted in lysis buffer and 35 µL NuPage SDS sample buffer to a protein concentration of 50 µg protein in 100 µL loading solution. Precisely, 10 µL reduced samples (5 µg protein) per lane were run on a NuPage 1.0-mm 15 well 4–12% Bis-Tris gel and transferred onto PVDF membranes (Invitrogen, Carlsbad, CA, USA). The transferred PVDF membranes were incubated in 5% skimmed milk in PBS containing 0.05% Tween 20 (PBST) for blocking, and then were incubated with primary antibody diluted at 1:10,000 in PBST. PVDF membranes were washed four times for 15 min in PBST, incubated with HRP-conjugated secondary antibody diluted at 0.08 μg/mL in PBST for 30 min, and washed 4 × 15 min in PBST. Signals were visualized using Western Lightning ECL (Pierce, Rockford, IL, USA) and Biomax XAR film (Carestream Health Inc, Rochester, NY, USA). Protein band intensity was analyzed by ImageJ (NIH, Bethesda, MD, USA).

### 2.3. Cell Immunofluorescence

AQP2-MDCK cells were plated on coverslips and incubated for 3 days to form polarized confluent monolayers. The cells were treated with CPZ (100 µM) for 15 min combined with or without FK (10 µM) for 10 min and fixed in 4% PFA at 37 °C for 10 min. For cold shock experiments, cells were grown on polyester filters for 4 days; culture medium was replaced with ice-cold DMEM, and cells were incubated at 4 °C for 15 min before fixation in 4% PFA at 4 °C for 30 min. After PFA fixation, cells were washed three times with PBS, permeabilized with 0.1% Triton-X100/PBS for 10 min, treated with 0.5% SDS/PBS for 5 min, and blocked in 1% BSA/PBS for 30 min at room temperature. Primary antibodies used were: anti-AQP2 (0.4 μg/mL), alpha-tubulin (4.8 μg/mL), and clathrin (4.8 μg/mL) in 1% BSA/PBS. They were added on both sides of the membrane and incubated overnight at 4 °C in a humid chamber. Cells were washed in PBS (10 min, three times), incubated with diluted secondary antibody (2.4 μg/mL) for 3 h at room temperature, and then washed in PBS (10 min, three times). For F-actin staining, 20 nM rhodamine phalloidin diluted with PBS was applied to PFA-fixed cells that were permeabilized with PBS containing 0.1% Triton X-100 but no SDS, which we found reduces the phalloidin/actin interaction. After washing, coverslips were mounted on glass slides with Vectashield Mounting Medium containing DAPI (Vector Labs, Burlingame, CA, USA) diluted 2:1 in 0.1M Tris-HCl, pH 8.0. The mounted slides were examined using a Zeiss 63 × 1.4NA Plan Apo objective mounted on a Zeiss LSM800 confocal microscope (Carl Zeiss MicroImaging Inc., Thornwood, NY, USA). All images were acquired within the linear range of intensity, and no thresholding was applied to the images.

### 2.4. Transferrin Endocytosis Assay 

The AQP2-MDCK cells were grown on filters for more than 4 days to form polarized confluent monolayers. The cells were treated with CPZ (100 µM) for 15 min or MBCD (10 mM) before addition of rhodamine-tagged transferrin (Rho-Tf: 25 μg/mL) to the basolateral side of the cells for 30 min at 37 °C. After incubation, the cells were washed 2 × 5 min with PBS and then reincubated at 37 °C in DMEM medium with or without CPZ. After 10 min incubation, cells were fixed in 4% paraformaldehyde containing 5% sucrose. The cell plasma membranes were stained with wheat germ agglutinin conjugated to Alexa Fluor 647 (0.4 μg/mL) for 10 min. After washing, coverslips were mounted on glass slides with Vectashield mounting medium containing DAPI for nuclear staining. The mounted slides were examined using a Nikon A1R confocal microscope (Nikon Instruments, Tokyo, Japan). Images were quantified using a method similar to that we have previously described [36]. In brief, the cell membranes were visualized by staining with Alexa Fluor 647 conjugated to wheat germ agglutinin (0.4 ug/mL) (Invitrogen) for 10 min. The nucleus was costained when mounted with Vectashield containing DAPI. The fluorescence channel corresponding to the Rho-Tf was shutoff, and the region of interest (membrane and the cytoplasmic areas) were traced using the fluorescence labeling by the Alexa Fluor 647-conjugated wheat germ agglutinin signal. The fluorescence in the Rho-Tf channel was then activated, and the fluorescence intensities were evaluated in the membrane and the cytoplasmic regions determined by using Alexa Fluor 647-conjugated wheat germ agglutinin and corrected for nonspecific labeling by subtraction of the background fluorescence observed in the nucleus, delimited by using DAPI. The quantification was performed on 30 cells in each condition from 5 different images. The mean fluorescence intensity of the plasma membrane area from 30 cells for each sample was quantified using Volocity software (Quorum Technologies, Puslinch, Canada).

### 2.5. In Situ Kidney Tissue Slices

The effect of CPZ on AQP2 trafficking was studied in kidney tissue slices incubated in vitro as previously reported [36,37,38]. All procedures described were reviewed and approved by the Massachusetts General Hospital (MGH) Subcommittee on Research Animal Care and were performed in accordance with the National Institutes of Health Guide for the Care and Use of Laboratory Animals. Briefly, Sprague-Dawley rats (250 g) were anesthetized using isoflurane inhalation. The kidneys were perfused using HBSS (110 mM NaCl, 5 mM KCl, 1.2 mM MgSO_4_, 1.8 mM CaCl_2_, 4 mM sodium acetate, 1 mM Na_3_ citrate, 6 mM D-glucose, 6 mM L-alanine, 1 mM NaH_2_PO_4_, 3 mM Na_2_HPO_4_, and 25 mM NaHCO_3_; pH 7.4) at 37 °C equilibrated with 5% CO_2_ and 95% O_2_. Both kidneys were cut into 2–3 mm-thick slices using a razor blade and then rapidly sliced with a Stadie-Riggs microtome (Thomas Scientific, Swedesboro, NJ, USA) into 0.5 mm slices. All of the slices were incubated at 37 °C for 15 min in equilibrated HBSS before treatment with drugs diluted in HBSS. Slices were incubated for 10 or 15 min with AVP (100 nM) or for 15 and 60 min with CPZ (400 μM). After stimulation, all of the slices were fixed by immersion in paraformaldehyde-lysine-periodate (PLP) fixative at room temperature for 20 min, and then vials were stored overnight in fixative at 4 °C. The slices were then rinsed three times in 10 mM sodium phosphate buffer containing 0.9% NaCl, pH 7.4, and stored in the same buffer plus 0.02% NaN_3_ at 4 °C. For immunostaining, the kidney slices were first incubated overnight in PBS containing 30% sucrose. The cryoprotected slices were mounted on a cutting block covered with OCT Compound 4583 (Tissue-Tek; Miles Inc., Elkhart, IN, USA). The frozen tissues were cut into 5 μm sections using a Leica CM3050S Cryostat (Leica, Buffalo Grove, IL, USA). The sections were attached to Superfrost-Plus glass slides (Fisher Scientific, Pittsburgh, PA, USA), then rehydrated in PBS for 20 min, treated with 1% SDS in PBS for 5 min as an antigen-retrieval step as previously described (36-38) and washed three times with PBS. The sections were then incubated 20 min in PBS containing 1% bovine serum albumin as a blocking step, and incubated overnight at 4 °C with AQP2 antibodies diluted (0.4 μg/mL) in PBS. After incubation, sections were washed three times in PBS. The secondary antibody, a donkey anti-goat IgG conjugated to Alexa fluor 488 diluted in PBS (7.5 μg/mL; Jackson ImmunoResearch, West Grove, PA, USA) was incubated 3 h at room temperature. Some sections were costained for clathrin. Sections were incubated with the anticlathrin antibody (0.5 μg/mL) for 1 h and then washed three times with PBS buffer. Clathrin was detected using a CY3-conjugated donkey antimouse IgG (1.9 μg/mL; Jackson ImmunoResearch, West Grove, PA, USA). For F-actin costaining, the AQP2-stained sections were incubated with 20 nM rhodamine phalloidin diluted with PBS for 30 min. After costaining with either clathrin or F-actin, the sections were mounted on glass slides with Vectashield mounting medium diluted 2:1 in 0.1M Tris-HCl, pH 8.0. Importantly, we found that pretreatment of sections with SDS reduced the ability of rhodamine phalloidin to interact with actin, so this step was omitted when actin visualization was required. The mounted slides were examined using a Zeiss LSM800 confocal microscope (Carl Zeiss MicroImaging Inc., Thornwood, NY, USA). The quantification of F-actin was performed on 30 cells in each condition from 3 different rats. The mean fluorescence intensity of the apical and basolateral plasma membrane area from 30 cells as well as the total F-actin in the cell for each sample was quantified using Volocity software.

### 2.6. F-Actin Quantification

MDCK cells (8 × 10^4^) were plated on 24 well plates and incubated in normal DMEM for 3 days. DMEM was replaced with HBSS, and cells were incubated for 2 h. After treatment with latrunculin B or CPZ, HBSS buffer was removed and cells were incubated in binding buffer containing phalloidin (20 mM KH_2_PO_4_, 10 mM PIPES, 5 mM EGTA, 2 mM MgCl_2,_ 4% PFA, 0.1% Triton X-100, and 250 nM rhodamine phalloidin) for 15 min at room temperature. Negative controls to estimate background autofluorescence were prepared using binding buffer lacking rhodamine phalloidin. Cells were washed four times in PBS and incubated overnight in 300 µL (in each well) methanol at −20 °C to extract bound rhodamine phalloidin. The extracted rhodamine phalloidin fluorescence was read using a Beckman DTX-880 multiplate reader (excitation 535 nm and emission 595 nm). F-actin content values were expressed as relative fluorescence after subtraction of the negative-control values. Data were adjusted for protein concentration. Each fluorescence value was expressed as relative fluorescence unit (RFU)/protein (µg), and data were analyzed using one-way ANOVA.

### 2.7. Statistical Analyses

Data were expressed as the mean ± SD. Statistical significance was evaluated using one-way ANOVA or *t*-test with Welch’s correction when applicable.

## 3. Results

### 3.1. AQP2 Accumulates in the Basolateral Plasma Membrane After CPZ Treatment

**Figure 1:**
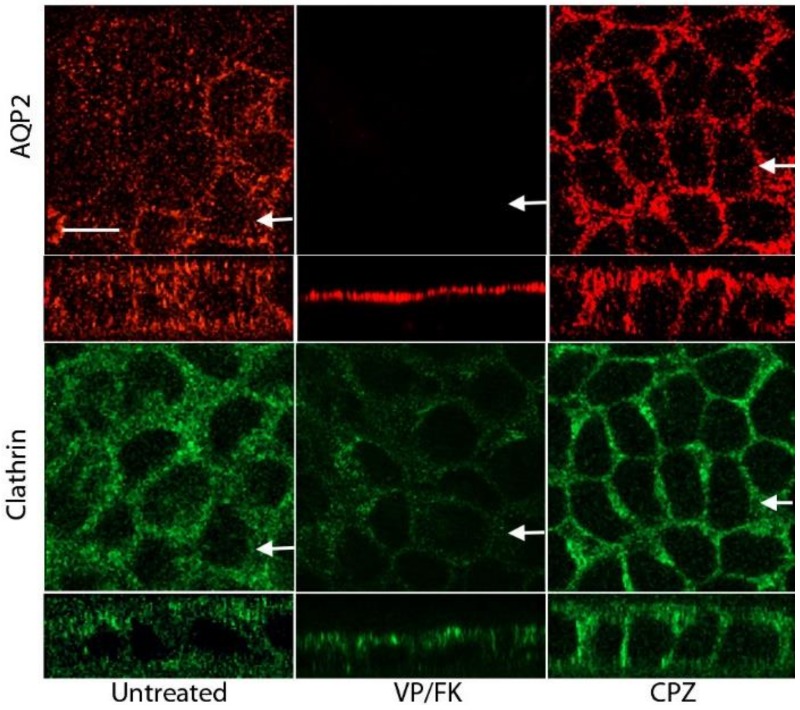
Chlorpromazine (CPZ) causes basolateral redistribution of aquaporin-2 (AQP2) in polarized Madin–Darby canine kidney (MDCK) cells: AQP2-MDCK cells were untreated (left panels) or treated with either vasopressin (VP)/forskolin (FK) (middle panels) or CPZ (CPZ, 100 µM for 15 min) (right panels). After treatment, AQP2 or clathrin were immunodetected. The larger panels represent confocal sections through the middle region of the cell monolayer while the smaller horizontal strips at the bottom of each panel are Z-sections (taken in the plane indicated by the white arrows) that show apical and basolateral membranes. VP/FK treatment resulted in apical redistribution of AQP2 and clathrin, whereas after CPZ treatment, basolateral AQP2 and clathrin signals were clearly increased. The images are representative of three independent experiments. Bar = 10 µm (for all panels).

Under normal incubation conditions, AQP2 accumulates in the apical plasma membrane of MDCK cells after vasopressin and forskolin treatment, with selective apical F-actin depolymerization [21,39,40], as described in mammalian kidney principal cells in situ [41]. Without CPZ treatment, AQP2 was localized mainly in the perinuclear region (Figure 1, upper left panels), while in the presence of VP/FK, AQP2 is mainly apical (Figure 1, upper middle panels). In contrast, AQP2 accumulated in the basolateral plasma membrane after CPZ treatment (Figure 1, upper right panels). Similar to AQP2, clathrin was also concentrated in the perinuclear area under control conditions (Figure 1, left panels), and at the apical pole of the cells after VP/FK treatment (Figure 1, middle panels), but accumulated in the basolateral region after CPZ treatment (Figure 1, right panels). These data suggest that AQP2 is targeted to the basolateral plasma membrane, poised to be retrieved by clathrin-mediated endocytosis in MDCK cells. This finding is similar to our previous data obtained using a brief cold shock to block AQP2 in the basolateral membrane [30].

### 3.2. CPZ Does Not Modify AQP2 Phosphorylation 

**Figure 2:**
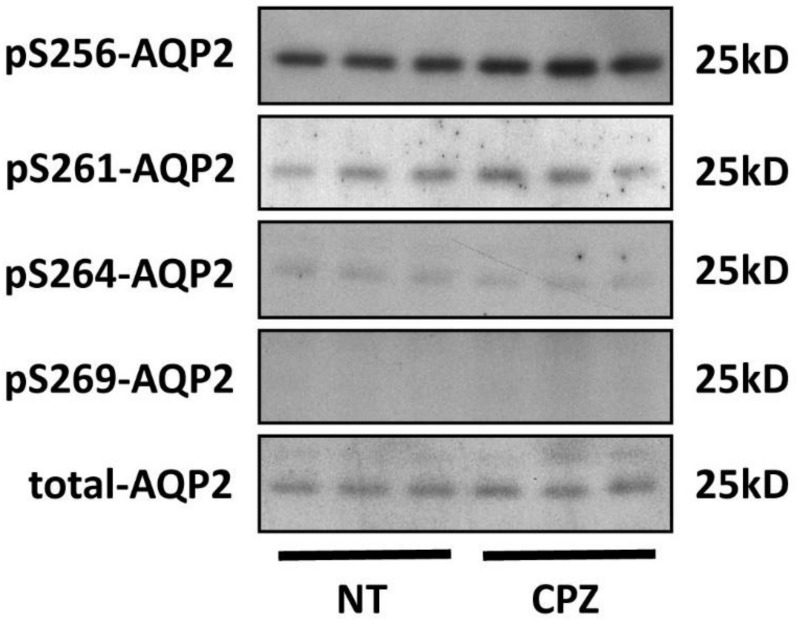
CPZ does not affect AQP2 phosphorylation in polarized MDCK cells: AQP2-MDCK cells were incubated with CPZ (100 µM for 15 min) or without CPZ (NT), then were subjected to Western blotting. After CPZ treatment, AQP2 phosphorylation status at Ser-256, Ser-261, Ser-264, and S269 was not changed.

We then asked whether CPZ-mediated basolateral accumulation is dependent on the phosphorylation state of AQP2. After CPZ treatment, no significant changes in phosphorylation at Ser-256, Ser-261, Ser-264, and Ser-269 were detected (Figure 2). The specificity of these antibodies was previously verified using vasopressin and forskolin treatments [30]. These results indicate that CPZ-induced basolateral accumulation of AQP2 is independent of changes in its overall phosphorylation state.

### 3.3. CPZ Inhibits Clathrin-Mediated Endocytosis of Transferrin in MDCK Cells

**Figure 3:**
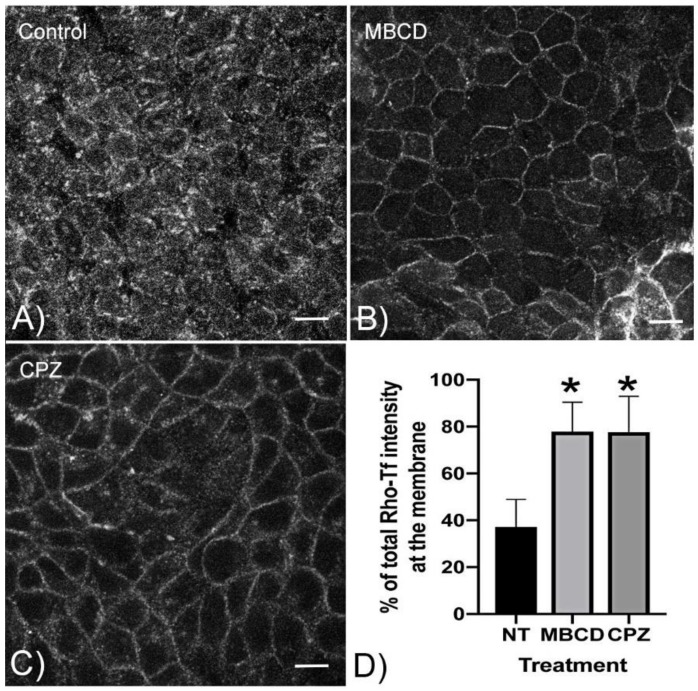
**CPZ blocks endocytosis in AQP2-MDCK cells:** Polarized AQP2-MDCK cells were grown on filters for 4 days (**A** to **C**). Cells were preincubated with methyl-β-cyclodextrin (MBCD) (10 mM), a nonspecific endocytosis inhibitor, or CPZ (100 µM) for 15 min before the addition of Rhodamine-transferrin (Rho-Tf) (25 µg/mL) to the basolateral side of the cells. After incubation and fixation, Rho-Tf is scattered in vesicles throughout the cytoplasm in the untreated cells (**A**), whereas the Rho-Tf accumulates at the plasma membrane in cells treated with MBCD (**B**) or CPZ (**C**). Images were acquired using a Nikon A1R confocal microscope. These images are representative of three independent experiments. All images were taken at a central Z-axis plane that contained the nucleus of the cells (Bars = 10 µm). Quantification of the images was performed using Volocity software (**D**). The plasma membrane was identified by WGA-Alexa Fluor 647 staining. The intensity of Rho-Tf in the membrane and the cytoplasmic areas was analyzed by manually defining regions of interest. The fluorescence in the nucleus was used as a background level that was subtracted from all other values. At least 50 cells were analyzed in each of the three sets of images. The result is expressed as the % of total Rho-Tf fluorescence intensity at the membrane. The result is expressed as the mean ± SD, and data were analyzed using one-way ANOVA multicomparison (*p < 0.001, *n* = 3).

To show directly that CPZ inhibits endocytosis in MDCK cells, we examined the effect of CPZ on the cellular uptake of transferrin, a ligand that is known to be internalized by clathrin-mediated endocytosis (Figure 3A–D). In the absence of drugs, transferrin is endocytosed into the cells as expected (Figure 3A). In contrast, when cells are treated with MBCD, a nonspecific endocytosis blocker, or with CPZ, transferrin accumulates at the basolateral cell surface and shows considerably less internalization (Figure 3B,C). Quantification of Rho-Tf fluorescence intensity in the cells treated with MBCD or CPZ shows a significant reduction of Rho-Tf internalization and a significant plasma membrane accumulation of Rho-Tf (Figure 3D). 

### 3.4. CPZ Causes Basolateral Accumulation of AQP2 and Clathrin in Kidney Slices

**Figure 4:**
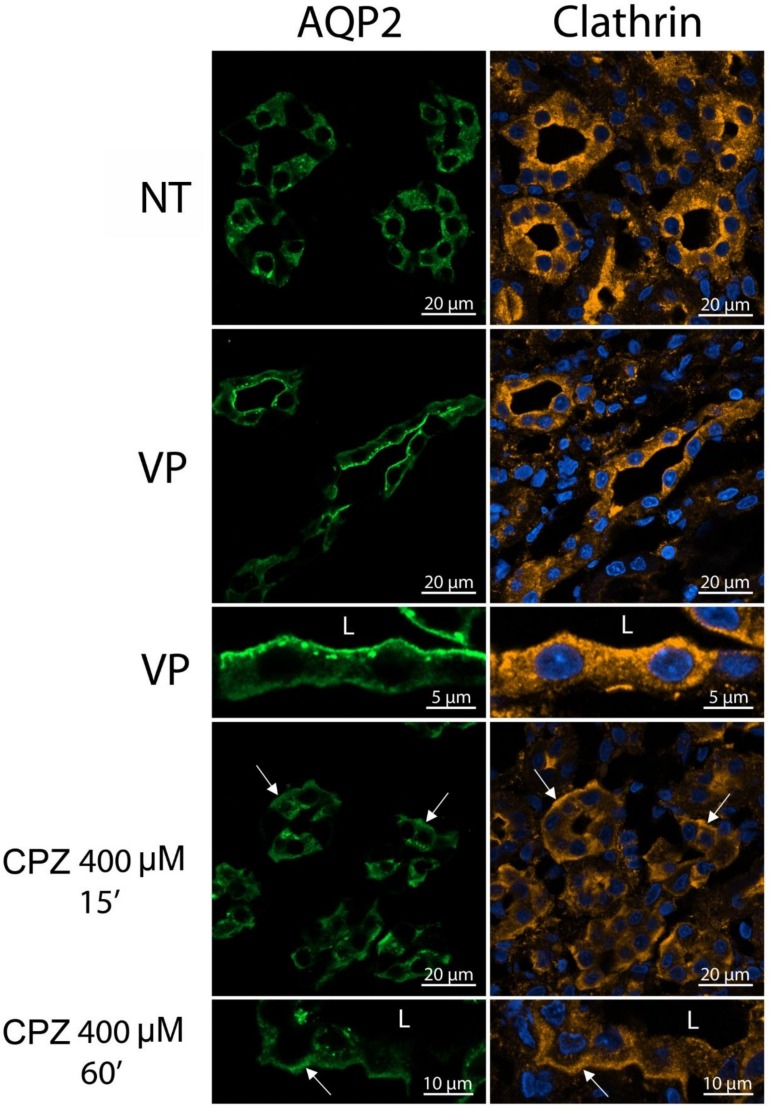
CPZ affects both clathrin and AQP2 subcellular distribution in rat kidney slices. Rat kidney slices were incubated with or without CPZ (400 µM for 15 min), and then, AQP2 and clathrin were immunolocalized. To test cell viability, some slices were treated with vasopressin (AVP, 100 nM for 10 min). After AVP treatment, the apical AQP2 signal was greatly increased in principal cells, with increased apical clathrin signals also (middle panels). In contrast, after CPZ treatment, the basolateral AQP2 signal was markedly increased along with the basolateral clathrin signal (lower panels).

Since CPZ affects clathrin and endocytosis in MDCK cells, we then examined the effect of CPZ on clathrin distribution in rat kidney slices in vitro. Without treatment, AQP2 was located at intracellular sites with some in the apical plasma membrane (Figure 4, green upper panels). Apical accumulation of AQP2 was increased by short-term incubation with AVP (100 nM, 10 min) (Figure 4, middle panels). In contrast, after CPZ treatment (400 µM, 15 min), the basolateral AQP2 signal was clearly increased together with an increased basolateral accumulation of clathrin (Figure 4, red, lower panels). 

### 3.5. CPZ Decreases Basolateral F-Actin Staining in MDCK Cells 

**Figure 5:**
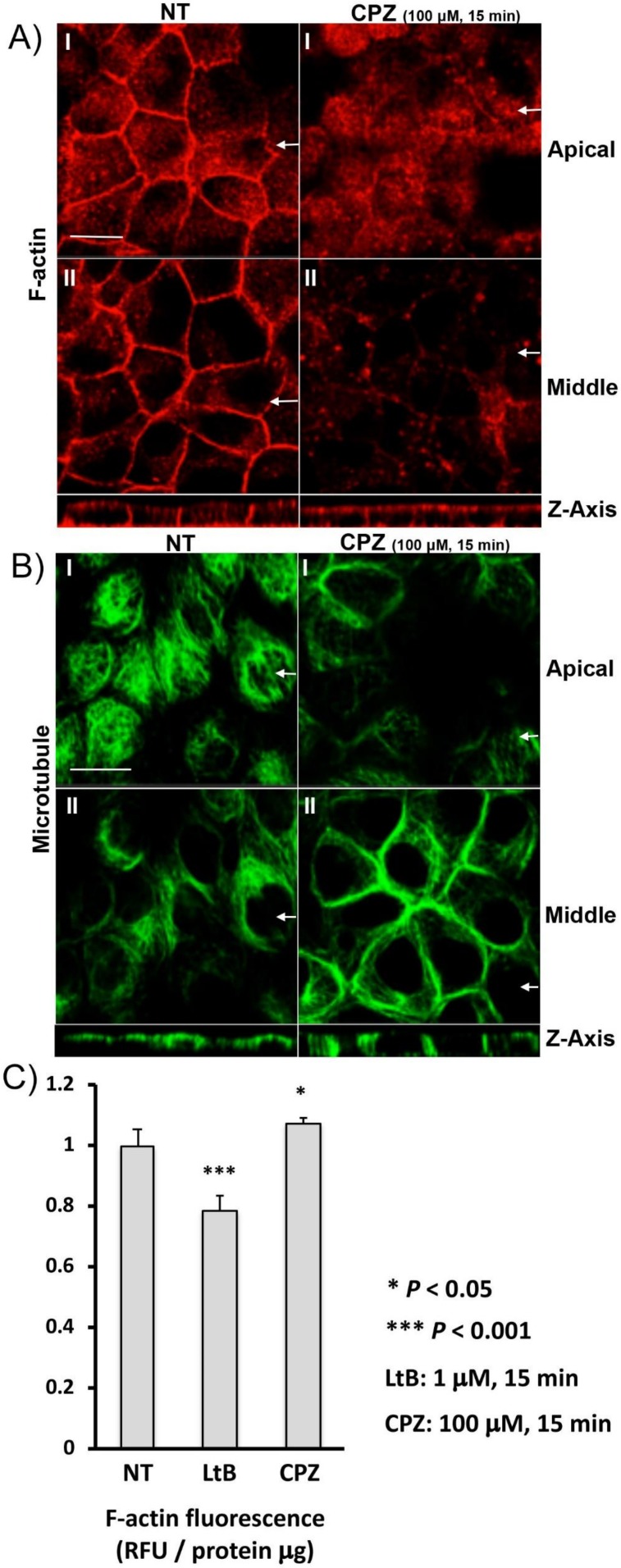
CPZ selectively disrupts basolateral actin but increases basolateral tubulin in polarized MDCK cells: (**A**) AQP2-MDCK cells were treated 15 min with 100 µM CPZ (right panels) or without CPZ (NT, left panels). F-actin was visualized using rhodamine phalloidin. The larger panels represent confocal sections of the apical (‘) and the middle region (“) of the cell monolayer. The smaller horizontal strips at the bottom of each column are Z-sections to show apical and basolateral membranes (taken in the plane indicated by the white arrows). After CPZ treatment, basolateral F-actin was selectively decreased but, in contrast, the apical actin signal was increased. The images are representative of three independent experiments. Bar = 10 µm (all panels). (**B**) AQP2-MDCK cells were treated 15 min with 100 µM CPZ (right panels) or without CPZ (NT, left panels) and then microtubules were visualized using an alpha-tubulin antibody. The larger panels represent confocal sections of the apical (‘) and the middle region (“) of the cell monolayer. The smaller horizontal strips at the bottom of each panel are Z-sections to show apical and basolateral membranes (taken in the plane indicated by the white arrows). After CPZ treatment, the basolateral microtubule (tubulin) signal was increased, but the apical signal was decreased. The images are representative of three independent experiments. Bar = 10 µm (all panels). (**C**) F-actin quantification assays were performed with or without CPZ treatment. AQP2-MDCK cells were treated with the F-actin depolymerizing drug latrunculin B (LtB, 1 µM for 15 min) or CPZ (100 µM for 15 min) and then were subjected to a rhodamine phalloidin based F-actin quantification assay. A significant 20% reduction in F-actin content was quantified after short-term LtB treatment (to 0.78 ± 0.05 of control levels, mean ± SD, *n* = 5, *p* < 0.001). In contrast, F-actin content was slightly but significantly increased after CPZ treatment (to 1.07 ± 0.02 of control levels, mean ± SD, *n* = 5, *p* < 0.05).

We then examined the effect of CPZ on F-actin organization by immunofluorescence using rhodamine phalloidin in MDCK cells. After CPZ treatment, the basolateral actin staining was decreased (Figure 5A), whereas the apical F-actin staining was increased. In contrast, microtubules detected by anti-alpha tubulin were more abundant in the basolateral regions after CPZ treatment (Figure 5B). We next quantified F-actin levels with or without CPZ treatment in MDCK cells. The validity of the quantification was tested using latrunculin B, a potent F-actin depolymerizing agent, which disrupts the actin network in MDCK cells as we have previously shown [21]. A significant 30% decrease of F-actin was quantified with short-term latrunculin B treatment (1 µM, 15 min, Figure 5C). In contrast, despite disruption of basolateral actin visualized by rhodamine phalloidin staining, there was a small overall increase in total cellular F-actin after CPZ treatment (Figure 5C).

### 3.6. CPZ Decreases Basolateral and Increases Apical F-Actin in Kidney Slices

**Figure 6:**
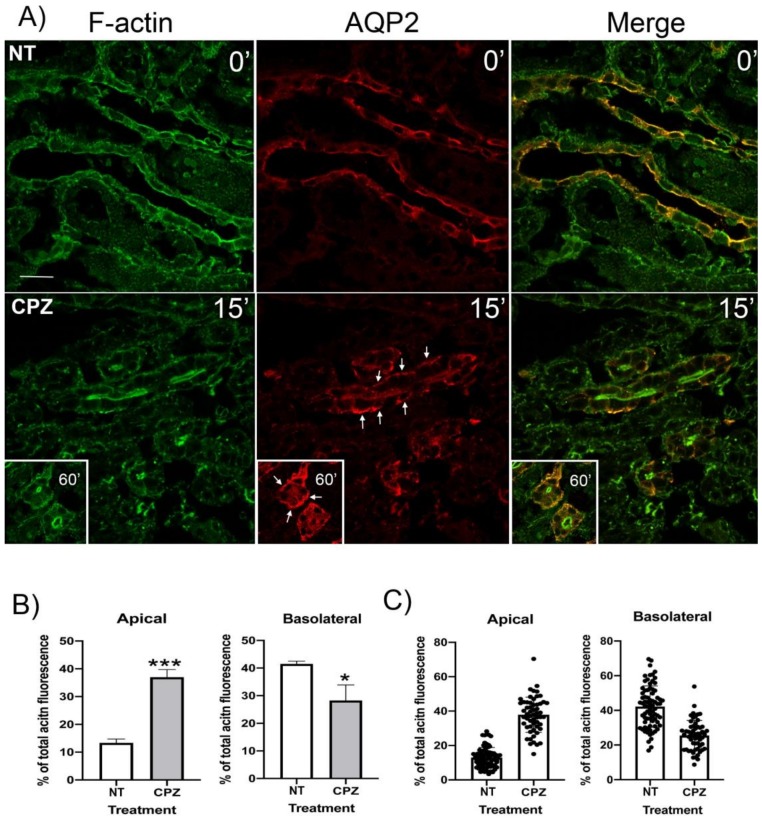
The effect of chlorpromazine on F-actin in rat kidney slices: rat kidney slices were treated with and without CPZ 400 µM 15 and 60 min (**A**) before fixation and immunostaining with anti-AQP2 antibody (red). F-actin was labeled with FITC-conjugated phalloidin (green). The basal condition (NT) showed apical or cytoplasmic distribution of AQP2 and F-actin in principal cells from the outer medulla. F-actin was also observed in the basolateral membrane. In contrast, CPZ treatment at both 15 and 60 min resulted in basolateral accumulation of AQP2 (arrows). F-actin appears more abundant in the apical pole than the basolateral pole in outer medullary principal cells. A comparison of F-actin distribution between the untreated condition and the 60 min CPZ treatment (inserts) shows a marked modification of F-actin distribution that favors apical membrane localization over basolateral localization (see quantification in **B** and **C**). The images are representative of three independent experiments (bar = 10 µm for all panels). Fluorescence intensity of F-actin at the apical and basolateral membranes was analyzed and expressed as the % of the total F-actin fluorescence in the cell (panels **B** and **C**). More than 20 cells were analyzed in 3 different animals and results were expressed as an average of each experiment (panel **B**; means ± SD, *n* = 3). They were analyzed using the Student *t* test (***p < 0.001 and *p < 0.05). In panel **C**, all of the individual measurements from the three stained tissues are illustrated together to show the spread in values among the conditions tested.

Next, we examined the effect of CPZ on F-actin in rat kidney slices. Under basal conditions (Figure 6, NT), AQP2 (red) is mainly apical in several cells and more cytoplasmic in others. The F-actin staining (green) shows an apical and basolateral signal (13.3 ± 1.4 vs. 41.5% ± 1.0%). However, after 15 min of exposure to CPZ, AQP2 (red) fluorescence intensity is increased by 195% ± 34% (*n* = 3) in the basolateral membrane region of the cells (Figure 6A). In parallel, basolateral F-actin is reduced to 28.3% ± 5.6% of the total F-actin fluorescence intensity while the amount of apical F-actin actually increases to 37.0 ± 2.7 (Figure 6B,C). The effect of CPZ also persists after 60 min of treatment as observed in the smaller insets.

### 3.7. Cold Shock Increases Basolateral AQP2 Without Depolymerizing F-Actin 

**Figure 7:**
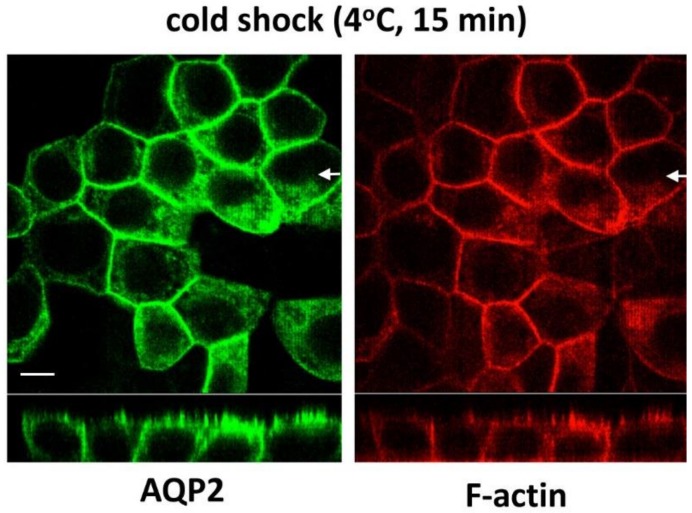
The effect of cold shock on F-actin in polarized MDCK cells: polarized AQP2-MDCK cells were subjected to a cold shock (4 °C for 15 min), and then, AQP2 and F-actin were immunostained. The larger panels represent confocal sections through the middle regions of each monolayer. The smaller horizontal strips at the bottom of each panel are Z-sections to show apical and basolateral membranes (taken in the plane indicated by the white arrows). After a brief cold shock, AQP2 accumulated in the basolateral membrane, with well-polymerized basolateral F-actin shown using Rh-phalloidin staining. The images are representative of three independent experiments. Bar = 5 µm.

To further delineate the function of basolateral F-actin for AQP2 intracellular trafficking, we examined the influence of a cold shock on basolateral F-actin organization. We previously reported that both AQP2 and clathrin accumulated in the basolateral plasma membrane after brief cold shock (4 °C for 15 min) [30]. AQP2-MDCK cells were incubated at 4 °C for 15 min in normal DMEM, fixed at 4 °C, and F-actin was stained with rhodamine phalloidin. Contrary to CPZ treatment, however, basolateral F-actin was not depolymerized during the cold incubation (Figure 7, right panel, red), but basolateral accumulation of AQP2 was still strongly induced (Figure 7, left panel, green). 

### 3.8. CPZ Inhibits Forskolin-Induced Apical Membrane Accumulation of AQP2

**Figure 8:**
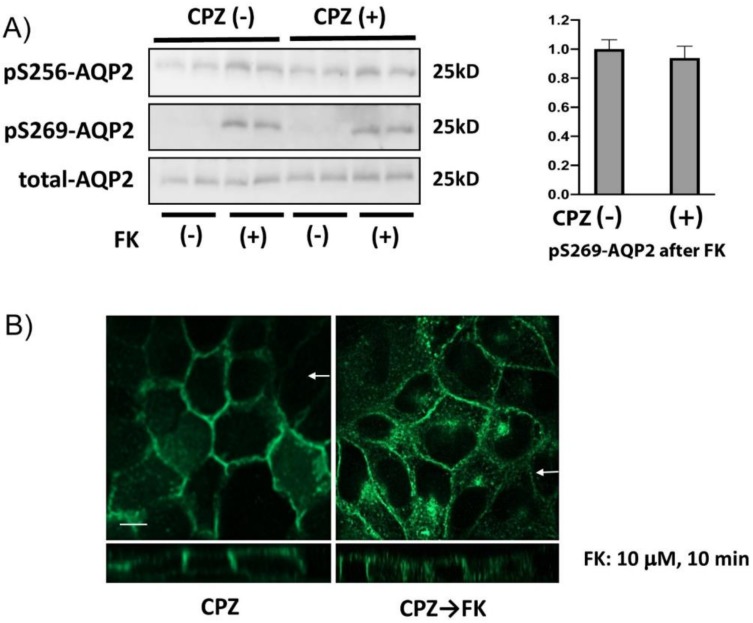
The effect of CPZ on FK-mediated AQP2 phosphorylation and translocation. (**A**) AQP2-MDCK cells were pretreated with or without chlorpromazine (CPZ, 100 µM for 15 min), stimulated with forskolin (FK, 10 µM for 10 min), and then were subjected to Western blotting. The FK-induced increase of AQP2 phosphorylation at Ser-269 was not significantly changed by CPZ pretreatment. Protein band intensity was analyzed by ImageJ. Ser-269-phosphorylated AQP2 signals adjusted by total AQP2 signals were analyzed by a two-tailed Student *t* test (mean ± SD, *n* = 4, NS = not significant). The images are representative of three independent experiments. (**B**) AQP2-MDCK cells were pretreated with CPZ (100 µM for 15 min), then treated with FK for a further 10 min (right panel) or without FK (left panel), and then immunostained to localize AQP2. The larger panels represent confocal sections through the middle regions. The smaller horizontal strips at the bottom of each panel are Z-sections to show apical and basolateral membranes (taken in the plane indicated by the white arrows). AQP2 remained basolateral or intracellular, and FK-induced apical translocation of AQP2 was not observed after CPZ pretreatment. The images are representative of three independent experiments. Bar = 5 µm.

Finally, we investigated the effect of CPZ pretreatment on FK-mediated AQP2 phosphorylation and intracellular translocation (Figure 8). The FK-induced increase of phosphorylation at Ser-269 was equally robust with or without CPZ pretreatment (Figure 8A). However, FK-induced apical AQP2 accumulation (as shown in Figure 1) was not detected; most of the AQP2 remained in the basolateral plasma membrane, with some intracellular, after CPZ pretreatment despite the presence of forskolin (Figure 8B). 

## 4. Discussion

The significant observation from this study is that CPZ induces basolateral AQP2 plasma membrane accumulation by blocking its clathrin-mediated endocytosis and subsequent delivery to an apical targeting pathway. In parallel, CPZ affects the cellular distribution of F-actin, resulting in a reduction of the level of basolateral F-actin, which may play a role in inhibiting AQP2 clathrin-mediated endocytosis. This trafficking itinerary occurs not only in renal epithelial cells in culture but also in kidney principal cells in situ.

The presence of AQP2 in the basolateral membrane of cells in culture and the kidney in situ has been described repeatedly [21,23,26,29,30]. The amount of AQP2 in the apical plasma membrane is a major rate-limiting factor that determines collecting duct water permeability in vivo, but the contribution of basolateral AQP2 to transepithelial water permeability is unclear because of the expression of two other basolateral aquaporins in principal cells, AQP3 and AQP4 [23]. Although the mechanism that regulates apical AQP2 membrane expression has been extensively studied, the exocytosis and endocytosis pathways that regulate basolateral membrane AQP2 expression have proven to be more elusive. In our previous study, however, we observed an increase of AQP2 accumulation in the basolateral membrane of MDCK cells after cooling cells to 4 °C for a few minutes [30]. This maneuver appears to slow down basolateral membrane AQP2 endocytosis sufficiently to allow its accumulation in this membrane domain [30]. Under normal circumstances, AQP2 is rapidly internalized following transient basolateral insertion, and delivered to apical recycling endosomes using a microtubule-dependent pathway [30].

Interestingly, we now show that CPZ treatment also increases the accumulation of AQP2 in the basolateral membrane both in MDCK cells as well as in rat principal kidney cells under normal physiological temperature conditions (37 °C). While the role of AQP2 in basolateral membrane permeability is unclear, it may play a role in epithelial morphogenesis by interacting with integrins and the extracellular matrix, as suggested by Chen et al. [29]. This result suggests that CPZ is a useful tool to understand the events that lead to basolateral membrane AQP2 accumulation. CPZ, an antipsychotic medication, is a dopaminergic antagonist [42] and a calmodulin inhibitor [43]. CPZ also affects redistribution and the assembly of clathrin-coated pits [44,45,46] and can inhibit actin polymerization [47], both of which are critically involved in AQP2 trafficking [20,21,41]. 

Actin depolymerization is necessary for apical AQP2 membrane accumulation to occur upon vasopressin stimulation. It has been suggested that F-actin filaments are a barrier that prevents AQP2 membrane accumulation by blocking the trafficking pathway of apically directed vesicles [16,48,49,50,51,52,53]. However, in our current study, AQP2 accumulated in the basolateral membrane even when basolateral F-actin was apparently not depolymerized at 4 °C, and AQP2 was nonetheless trapped in the basolateral membrane due to the acute arrest of clathrin-mediated endocytosis. This result suggests that basolateral F-actin is not a critical functional barrier that blocks AQP2 targeting to the membrane. Instead, actin may serve a role in short-range linking of AQP2 trafficking from the basolateral membrane to the microtubule “highway” that directs vesicles to the apical pole of the cell. This link between the actin and the microtubule cytoskeleton has been established in other trafficking systems [54,55,56,57,58]. In our previous report, we demonstrated that microtubules are indeed involved in AQP2 transport from the basolateral membrane. Briefly, VP/FK-induced AQP2 apical translocation during rewarming (after a brief cold shock) was related to microtubule reorganization; the process was inhibited by pretreatment with colchicine, a microtubule-depolymerizing agent [30]. These previous results are very similar to the effect of CPZ on FK-induced AQP2 translocation and together demonstrate that both intact basolateral F-actin and intact microtubules are necessary for the rapid AQP2 transcytosis from the basolateral membrane. It is reported that F-actin and microtubules are reciprocally interdependent. Such an F-actin function in basolateral AQP2 endocytosis might, therefore, be crucial for VP- and/or FK-induced AQP2 apical accumulation. Our results support the idea that apical and basolateral pools of F-actin are differentially regulated [59,60]. Furthermore, some actin-associated proteins such as PTEN and ERM (ezrin-radixin-moesin) are located only in the apical membrane, while phosphatidylinositol (3,4,5)-triphosphate accumulates in the basolateral membrane [61,62,63,64]. The specific membrane polarities of these molecules can modify and fine-tune the dynamics of actin polymerization. The differences in actin-organizing mechanisms between apical and basolateral sides of kidney epithelial cells may explain why plasma membrane accumulation of AQP2 is prolonged only in the apical plasma membranes after vasopressin treatment, while increased basolateral accumulation is associated with other conditions and treatments both in vivo and in vitro [24,26,30,65,66].

In this study, we also show that CPZ leads to an accumulation of clathrin in the basolateral membrane, which reflects an inhibition of clathrin-mediated endocytosis. Because CPZ inhibits the endocytosis of transferrin as previously described [44], we conclude that CPZ also inhibits the clathrin-mediated endocytosis of AQP2 at the basolateral plasma membrane [67]. The observed reduction of F-actin filaments in this region of the cell could be responsible for this effect. The interaction between F-actin and clathrin is, therefore, important for AQP2 endocytosis [49]. Indeed, we and others previously showed that disruption of actin organization using statins led to the apical membrane accumulation of AQP2 in vivo and to an increase in urine concentration ability in rodent models of diabetes insipidus [14,68].

In summary, our study confirms that the regulation of actin organization is different between the apical and basolateral sides of AQP2-expressing cells and that clathrin-mediated AQP2 endocytosis is blocked by CPZ at the basolateral pole. These data, using the drug CPZ, provide further evidence that AQP2 traffics through a transcytotic itinerary and show that this occurs not only in MDCK kidney epithelial cells in vitro but also in collecting duct principal cells in situ.
